# MiRNA-221-5p suppressed the Th17/Treg ratio in asthma via RORγt/Foxp3 by targeting SOCS1

**DOI:** 10.1186/s13223-021-00620-8

**Published:** 2021-12-04

**Authors:** Yuanyuan Guan, Yuemei Ma, Yao Tang, Xiaolei Liu, Yan Zhao, Lixin An

**Affiliations:** 1 Department of Allergy, First Affiliated Hospital of Harbin Medical University, 199 Dongdazhi Street, Nangang District, Harbin, 150001 Heilongjiang China; 2 Department of Allergy, The Second Affiliated Hospital of Harbin Medical University, Harbin, China

**Keywords:** Asthma, miRNA-221-5p, RORγt, SOCS1, Th17/Treg

## Abstract

**Background:**

This study was designed to investigate the mechanism and effects of miRNA-221-5p on the T-helper 17 (Th17)/T-regulatory (Treg) ratio in asthma.

**Methods:**

BALB/c mice were intranasally challenged with 100 µg OVA on 14 and 21 day. Mice were rechallenged with 2.5% OVA-PBS on 22 and 28 day. Mice were sacrificed using on day 30 under 35 mg/kg pentobarbital sodium. PBMCs were induced vitro model of asthma using 500 ng of lipopolysaccharides (LPS) for 4 h.

**Results:**

The expression of miRNA-221-5p was reduced in in vivo model, compared sham group. The *vitro* model of asthma treated with miRNA-221-5p mimic resulted in the reduction of IL-6, IL-17, IL-21 and IL-22 levels, and induction of IL-10, IL-35 and TGF-β levels. In addition, down-regulation of miRNA-221-5p induced the protein expression of suppressor of cytokine signaling 1 (SOCS1) and receptor-related orphan receptor-gamma-t (RORγt) and suppressed that of FOXP3 in in vitro model of asthma. Over-expression of miRNA-221-5p induced the protein expression of FOXP3, and suppressed that of SOCS1 and RORγt in in vitro model of asthma. The inhibition of SOCS1 or RORγt attenuated the effects of anti-miRNA-221-5p on Th17/Treg ratio in asthma.

**Conclusion:**

miRNA-221-5p may play critical roles in driving the differentiation of Th17/Treg ratio via RORγt/Foxp3 by Targeting SOCS1, reduced the function of Th17 cells by directly inhibiting RORγt/SOCS1 and promoted the function of Treg cells via Foxp3/ SOCS1 in asthma.

## Background

In recent years, bronchial asthma (asthma) has become a frequently-occurring and common disease in clinical practice due to changes in the environment and climate [1]. Asthma is mainly defined as a chronic inflammatory disease of the airways caused by various cells and cytokines [2]. At present, there are about 300 million patients with asthma globally [2]. According to certain statistical studies, there are approximately 30 million patients with asthma in China [2]. Moreover, with the climate and environmental changes in recent years, the prevalence of asthma in China has significantly increased, and the mortality rate has increased accordingly [3]. The main symptoms of asthma include paroxysmal wheezing, chest tightness, cough and shortness of breath, which occur and aggregate in the morning and/or at night [2]. The clinical cure rate of asthma is relatively low, with prolonged course of disease, which could easily lead to a variety of serious complications [3]. Untimely and irregular treatment is very likely to endanger patient lives, even seriously affecting their physical and mental health [3].

The monocyte proportion of peripheral blood helper T cells (Th17) in children with respiratory syncytial virus (RSV) bronchiolitis was higher than that in children with pneumonia and healthy children. However, the percentage of regulatory T cells (Treg cells) in CD4+ T cells in children with RSV bronchiolitis was lower than that of children with pneumonia and healthy children. These results suggest that Th17/Treg imbalance is related to the development of bronchiolitis [2, 4].

In recent years, Th17 cells have been demonstrated as a new subpopulation of CD4+ T cells, which could specifically produce cytokine IL-17. In addition, nuclear orphan receptor-γt (ROR-γt) is the specific transcription factor of Th17 cells. ROR-γt can induce the production and differentiation of Th17 cells, with a potent role in recruiting neutrophils. Therefore, Th17 cells can mediate the occurrence of airway inflammation in neutrophilic asthma [5].

Members of the suppressor of cytokine signaling (SOCS) protein family have also been confirmed to be involved in the occurrence and progression of multiple hypersensitivity reactions, autoimmune diseases and inflammatory diseases [6]. As important molecules, SOCS1 and SOCS3 have been indicated to affect the expression levels of multiple inflammatory factors and anti-inflammatory factors, including IL-6, IL-10, IL-12, IFN, LPS and etc., which can also suppress the signal transduction of various immune molecules [6]. To be specific, SOCS1 binds to JAK1, JAK2HE and JAK3 in Janus Kinases (JAKs) and inhibits their activities. JAKs/STAT signaling pathway plays an important role in the innate antiviral response of host. In addition, JAKs/STAT signaling pathway is considered as a cytokine signaling pathway with interferon function, which is a key factor mediating the antiviral and immunomodulatory effects of interferon [7].

microRNAs (miRNAs) are small non-coding single-stranded RNAs with 21 to 25nt in lengths. Studies have shown that there are 67 types of miRNAs with abnormal expression in asthma patients [8]. Additionally, these abnormally expressed miRNAs have up to 217 different types in asthma patients. miRNAs play an important role in development and inflammation in various diseases (such as cardiovascular disease, lung disease, tumors, etc.), which are also prominently expressed in rat models of asthma. Zhang et al. showed that miR-221-3p expression was significantly decreased in subjects with asthma [9]. In order to clarify further the molecular steps, this study investigated that the mechanism and effects of miRNA-221-5p on the Th17/Treg Ratio in asthma.

## Methods

### Sample collection

The study was carried out in strict accordance with the Guidelines For the Care and Use of Laboratory Animals of our hospital. 20 BALB/c mice aged 6–8 weeks and weighing 20–22 g were purchased from Laboratory Animal Center of our university. The mice were maintained in a specific pathogen-free room at 24 ℃ and 55–65% humidity, under a 12-h light/dark cycle with ad libitum access to standard chow and water. Mice were anaesthetized using 35 mg/kg pentobarbital sodium and sensitized by intraperitoneal injection of 10 µg ovalbumin (Sigma-Aldrich) and 1 mg aluminum hydroxide on days 0 and 7 day. Next, Mice were anaesthetized using 35 mg/kg pentobarbital sodium and intranasally challenged with 100 µg OVA on 14 and 21 day. Mice were rechallenged with 2.5% OVA-PBS on 22 and 28 day. Mice were sacrificed using on day 30 under 35 mg/kg pentobarbital sodium.

### Histological analyses and collection of BALF

Following sacrifice, tracheotomy was performed and 0.4 ml cold PBS was instilled into the lung. BALF was centrifuged at 250×*g* at 4 ℃ for 5 min and 100 µl BALF was used to measure the cytokine levels (TNF-α) using ELISA kits (H052-1, Nanjing Jiancheng Bioengineering Research Institute). Optical density (OD) value was detected using a microplate reader (Bio-Rad, Hercules, CA, USA) at the wavelength of 450 nm.

Lung samples were fixed with 4% paraformaldehyde, then embedded in paraffin. (10 μm thick) Sections was deparaffinized and rehydrated were stained with hematoxylin and eosin (H&E).

### Flow cytometric analysis

The spleen from each sacrificed mouse was removed, and splenocytes were prepared by gently crushing the tissues with a glass slide to release the cells. Cell was washed with PBS and 10% RPMI-1640 medium (Hyclone; GE Healthcare Life Sciences, Logan, UT, USA). Cell was stained with rat anti-mouse CD4 (FITC, 553046, 1:500, BD Biosciences, San Jose, CA, USA), allophycocyanin hamster anti-mouse CD3 (553066, 1:500, BD Biosciences, San Jose, CA, USA) at 4 ℃ for 30 min in darkness. Cell was stained with phycoerythrin (PE)-conjugated rat anti-mouse IL-17A antibody (559502, 1:300; BD Biosciences) at 4 ℃ for 30 min. Next, cell stained with FITC-conjugated rat anti-mouse CD4 (11-0042-81, 1:200; eBioscience, Inc.) and PE-conjugated rat anti-mouse CD25 (12-0251-81, 1:500; cat. no.) antibodies (eBioscience, Inc.) at 4 ℃ for 30 min for Treg cell. Cells were detected using a FACS Calibur flow cytometer (BD Bioscience) and analyzed using Flowjo 7.6.1 (FlowJo, LLC, Ashland, OR, USA).

### Quantitative real-time polymerase chain reaction (qRT-PCR) and microarray

Total RNA was extracted using Trizol reagent (Ambion, Austin, TX, United States) from from lung tissue samples or cell samples. RNA was reversely transcribed to complementary Deoxyribose Nucleic Acid (cDNA) using First Strand cDNA Synthesis Kit (Invitrogen, Carlsbad, CA, USA). *qRT-PCR was* performed using SYBRGreen miRNA assays (Genechem, Shanghai, China). The PCR primers used at Table 1. The relative expression levels of miRNAs were evaluated using the 2-ct method.

**Table 1:** qRT-PCR primer pairs

Name	Forward	Reverse
IL-6	5'-ACAACCACGGCCTTCCCTACT-3'	5'-CTCATTTCCACGATTTCCCAGA-3'
IL-17	5'-GCTGTTGCTGCTGCTGAG-3'	5'-TGGAACGGTTGAGGTAGTC-3'
IL-21	5'-GGACCCTTGTCTGTCTGGTAG-3'	5'-TGTGGAGCTGATAGAAGTTCAGG-3'
IL-22	5'-ATGAGTTTTCCCTTATGGGGAC-3'	5'-GCTGGAAGTTGGACACCTCAA-3'
IL-10	5'-GGACAACATACTGCTAACCGACTC-3'	5'-TTCATGGCCTTGTAGACACCTT-3'
IL-35	5'-TATGGTCAGCGTTCCAACAGC-3'	5'-TTCGGGACTGGCTAAGACACC-3'
TGF-β	5'-CAAACTAAGGCTCGCCAGTCC-3'	5'-TTGCGGTCCACCATTAGCAC-3'
β-actin	5'-GTGACGTTGACATCCGTAAAGA-3'	5'-GTAACAGTCCGCCTAGAACAC-3'
U6	5'-GCTTCGGCAGCACATATACTAAAAT-3'	5'-CGCTTCAGAATTTGCGTGTCAT-3'

Total RNA was extracted from cell samples and purified using the mir-Vana™ miRNA Isolation Kit. RNA assessed using an Agilent Bioanalyzer 2100 (Agilent Technologies, Santa Clara, CA, USA) and the Low Input Quick Amp WT Labeling Kit (cat.# 5190–2943; Agilent Technologies). Data was analyzed using the Gene Expression Wash Buffer Kit (cat.# 5188–5327; Agilent Technologies) and the Agilent Microarray Scanner (cat.#G2565CA; Agilent Technologies).

### Cell culture and transfection

Peripheral venous blood (10 ml) was collected form normal Volunteer. Peripheral blood mononuclear cells (PBMCs) were collected by Ficoll-Hypaque density gradient centrifugation. CD4+ T cells were separated using immunomagnetic beads, according to the manufacturer’s instructions (Miltenyi Biotec, Auburn, CA, USA). CD4+ T cells were cultured in Roswell Park Memorial Institute (RPMI)-1640 medium (without serum or antibiotics) containing 100 nM of the miRNA-221-5p mimic, miRNA-221-5p inhibitor or negative control mimics (GenePharma, Shanghai, China) using Lipofectamine 2000 (Invitrogen, Carlsbad, CA, USA) at 37 °C under a 5% CO2 for 4 h. After 4 h, old medium was removed and new medium was added into cell and cultivated at 37 °C under a 5% CO2 for 48 h. Cell was induced vitro model of asthma using 500 ng of lipopolysaccharides (LPS) for 4 h.

### Western blot

Protein extracts were prepared in lysis buffer with proteinase inhibitor from cell samples and quantified with BCA assay. 10 mg separated in 10% sodium dodecyl sulfatepolyacrylamide gel electrophoresis (SDS-PAGE) gels and then electroblotted onto a PVDF membranes. The membranes were blocked with 5% milk powder in Trisbuffered saline, probed with SOCS1, FOXP3, RORγt and GAPDH at 4 °C over-night. The membranes were washed with TBST and detected with a secondary antibody (Millipore, Billerica, MA, USA). Protein blanks were developed by enhanced chemiluminescence (ECL kit; Amersham) and analyzed using Image Lab 3.0 (Bio-Rad Laboratories, Inc.).

### Luciferase reporter assay

The sequence of the 3’-untranslated region (UTR) of SOCS1 was inserted the pGL3-CMV vector (Promega, Madison, WI, United States). HEK293T cells were transfected with the pGL3-basic construct along with either the miRNA- 221-5p mimic or negative control using Lipofectamine 2000 (Invitrogen). Cells were harvested, and luciferase activity was measured after 48 h.

### Immunofluorescence

After inducing LPS, PBMCs cells were washed with PBS and fixed with 4% paraformaldehyde for 20 min at room temperature. Cell was blocked with 5% BSA and 0.1% Trison X100 for 2 h at room temperature. Cell was probed with SOCS1 (sc-518028, 1:100, Santa Cruz Biotechnology) at 4 °C over-night. Cell was washed with PBS and probed with goat anti-rabbit IgG-CFL 555 sc-362272, 1:100, Santa Cruz Biotechnology) at 37 °C for 1 h. Cell was washed with PBS and stained with DAPI at 15 min in darkness. Cell was detected using the ChemiDoc XRS System (Bio-Rad, Hercules, CA, USA).

### Statistical analysis

We used Statistical Product and Service Solutions (SPSS) 19.0 software (IBM, Armonk, NY, USA) for statistical analysis. The *Student’st-test* was used for the comparison between two groups. The *one-way analysis of variance (ANOVA) and Tukey's post test* was used for the comparison between three groups. *p* < 0.05 was considered statistically significant.

## Results

### The expression of miRNA-221-5p in asthma

**Fig. 1:**
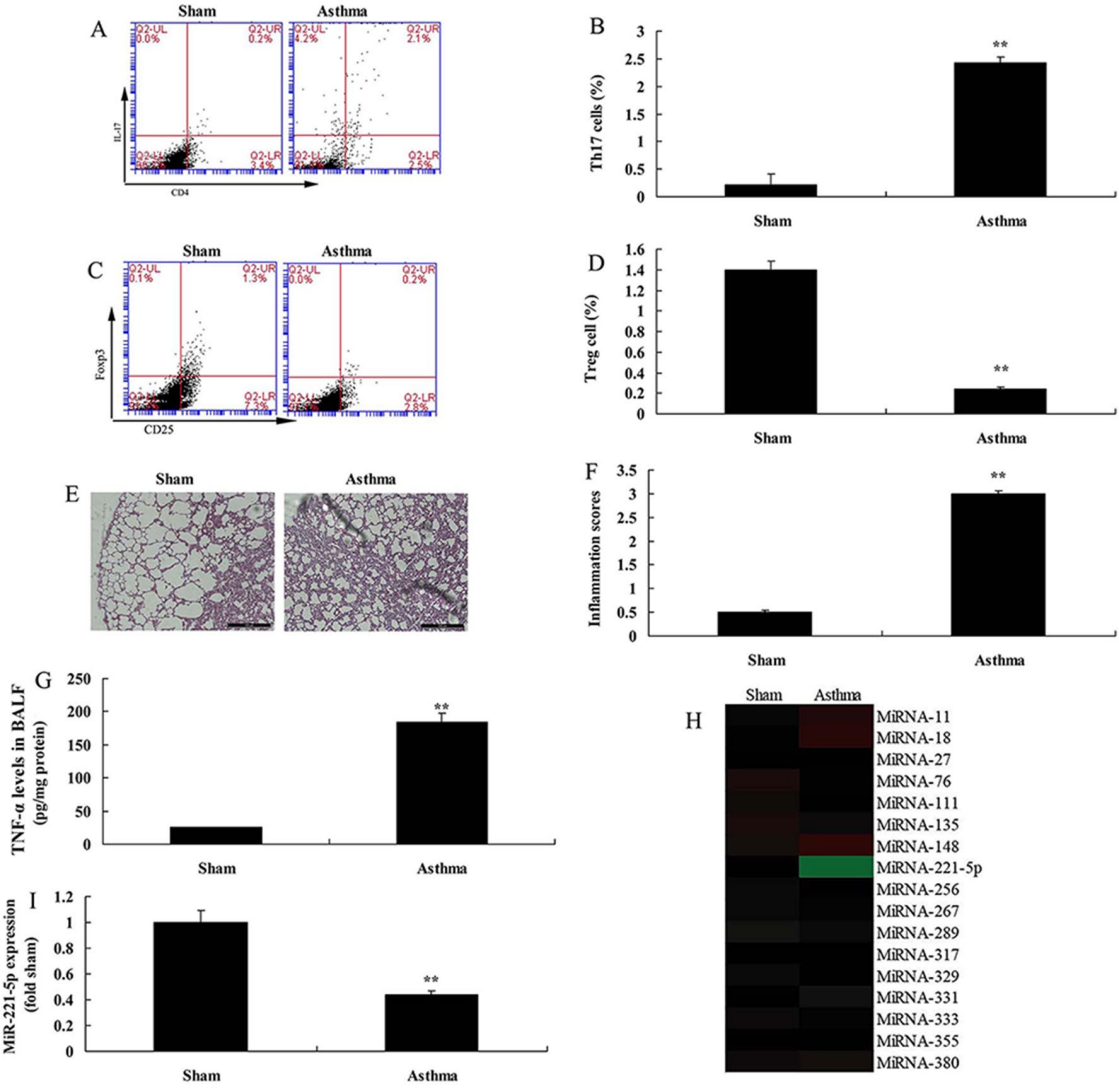
The expression of miRNA-221-5p in asthma. Th17 cell (**A** and **B**), Treg cells (**C** and **D**), HE staining (**E**), inflammation score (**F**), BALF levels (**G**), the expression of miRNA-221-5p in asthma by gene chip (**H**) and PCR (**I**). Sham, sham control group; Asthma, asthma model group. ^**^p < 0.01 versus sham control group

To explore the roles of miRNA-221-5p in Treg and Th17 cells ratio of asthma, the expression level of miRNA-221-5p was first measured in model of asthma. As a result, Th17 cells were increased while Treg cells were reduced in model of asthma, in comparison with sham group (Fig. 1A–D). Hematoxylin–eosin staining suggested that inflammation score was increased in model of asthma, compared with sham group (Fig. 1E–F). Moreover, the level of BALF cytokine was enhanced in model of asthma, in comparison with sham group (Fig. 1G). The expression of miRNA-221-5p from lung tissue samples was reduced in in vivo model of asthma, compared sham group (Fig. 1H–I). These results suggested that miRNA-221-5p might be able to regulate Treg and Th17 cells ratio of asthma.

### miRNA-221-5p regulates imbalance of Th17 and Treg Cells in model of asthma

**Fig. 2:**
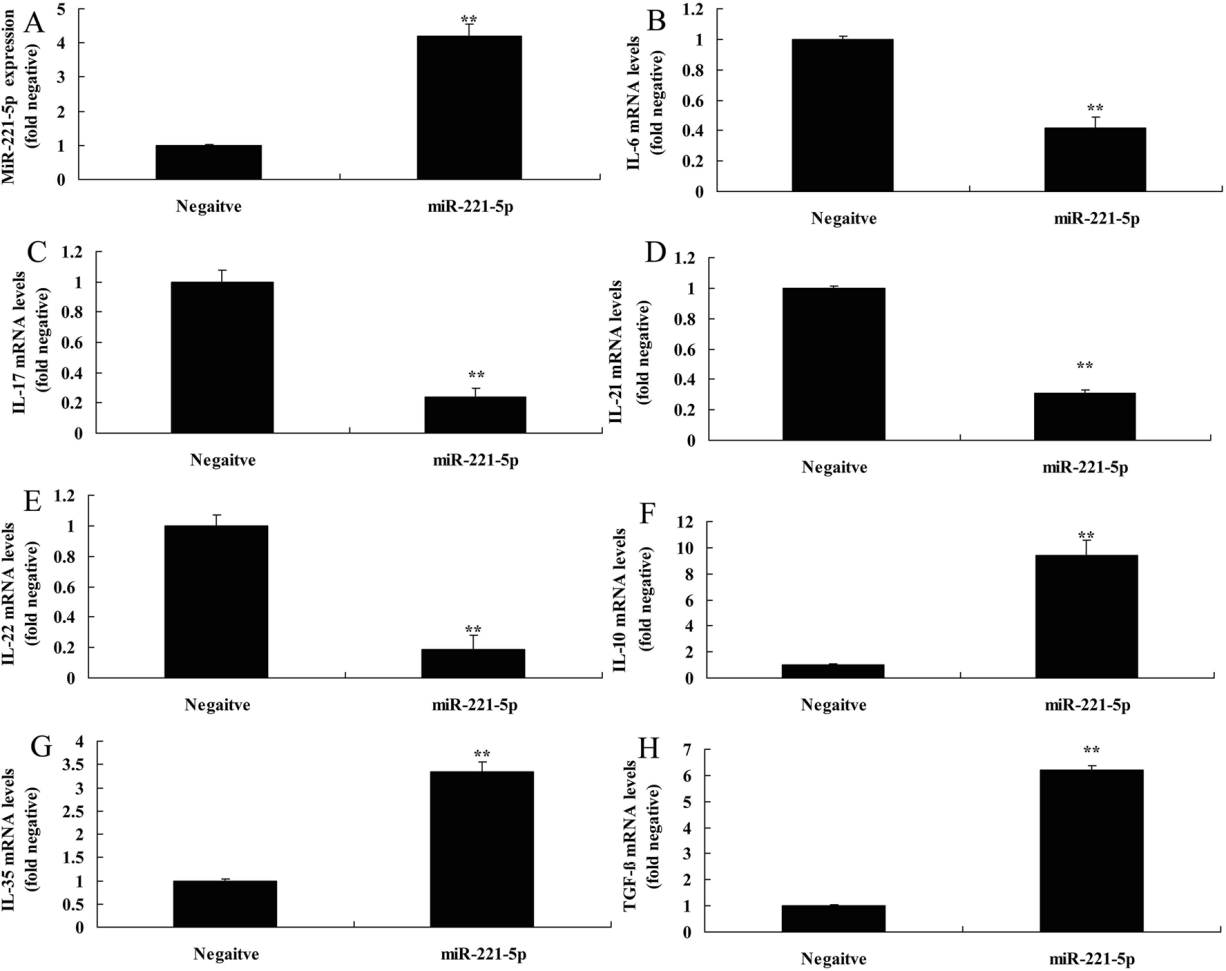
Over-expression of miRNA-221-5p regulates imbalance of Th17 and Treg Cells in model asthma. The expression of miRNA-221-5p (**A**), IL-6 (**B**), IL-17 (**C**), IL-21 (**D**), IL-22 (**E**), IL-10 (**F**), IL-35 (**G**) and TGF-β (**H**) levels. Negative, negative mimics group; miR-221-5p, Over-expression of miRNA-221-5p group. ^**^p < 0.01 versus negative mimics group

**Fig. 3:**
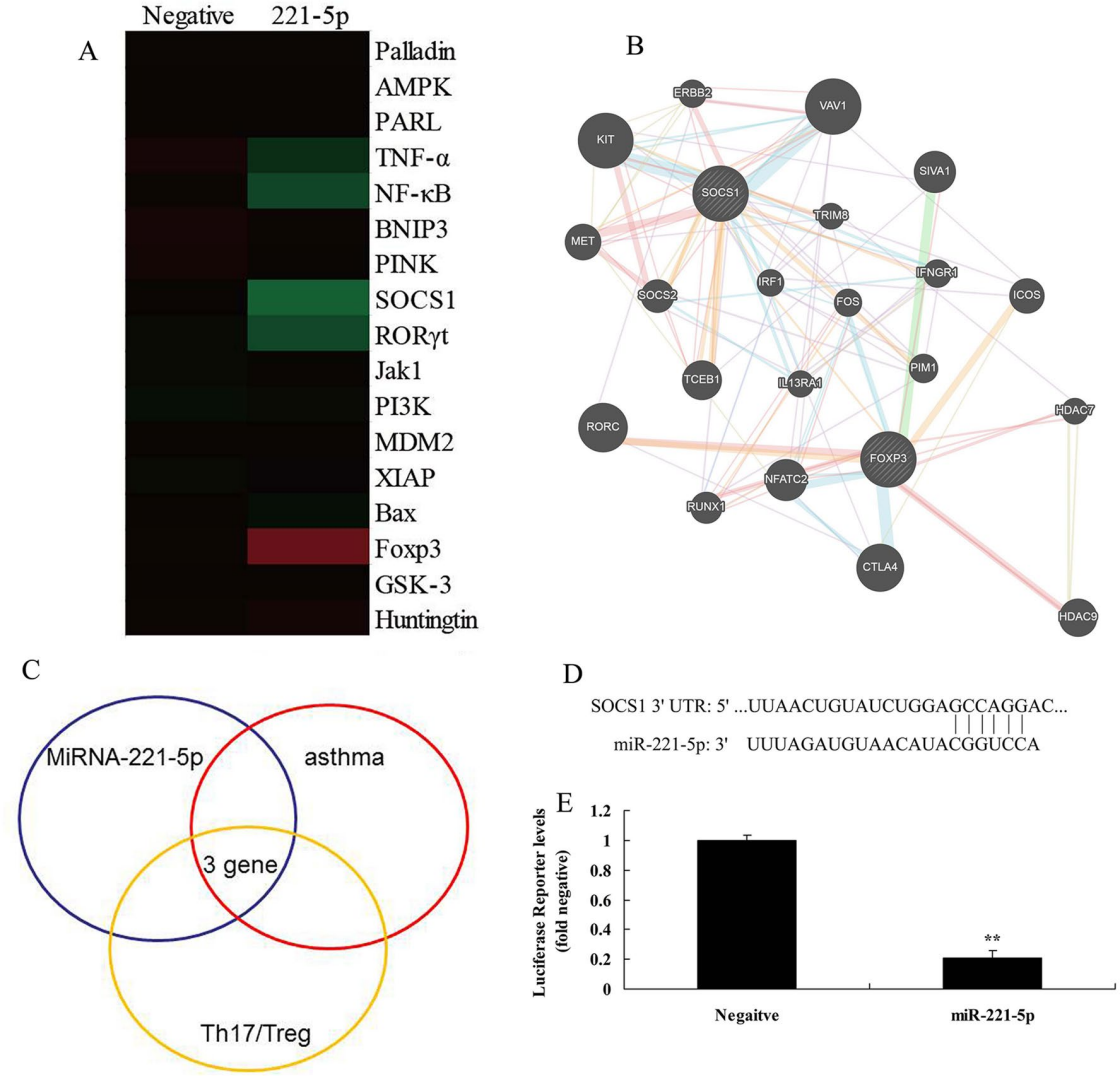
Over-expression of miRNA-221-5p regulates imbalance of Th17 and Treg Cells in model asthma through RORγt/Foxp3 by Targeting SOCS1. Heat map (**A**), network signal (**B**), Network signal analysis (**C**), 3’UTR region of SOCS1 showed potential alignment with miRNA-221-5p sequence (**D**), luciferase reporter activity levels (**E**). Negative, negative mimics group; miR-221-5p, Over-expression of miRNA-221-5p group. ^**^p < 0.01 versus negative mimics group

**Fig. 4:**
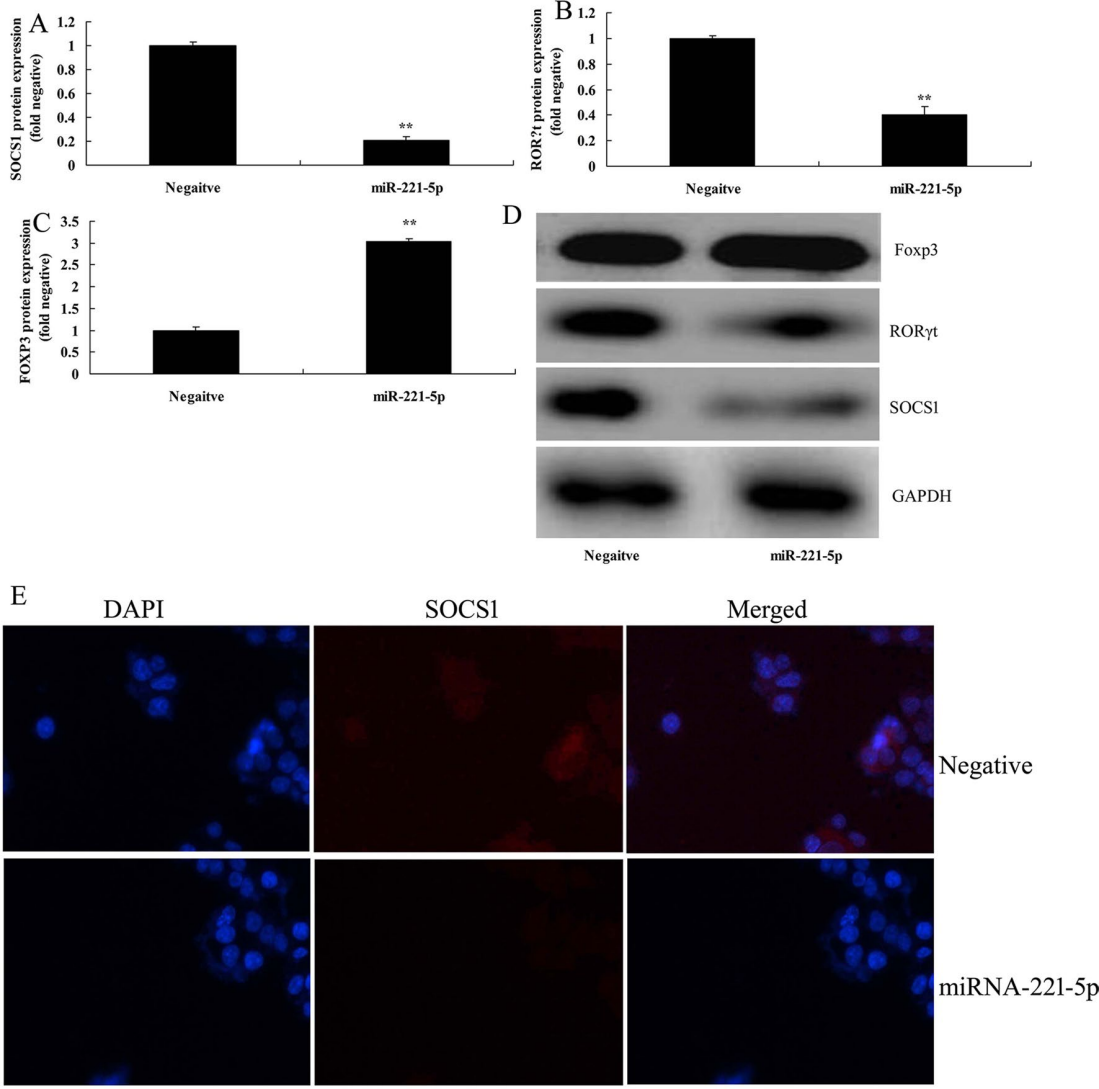
Over-expression of miRNA-221-5p regulates SOCS1, RORγt and Foxp3 expression. SOCS1, RORγt and Foxp3 protein expression by statistical analysis (**A**–**C**) and western blotting analysis (**D**). SOCS1 expression using Immunofluorescence (**E**). Negative, negative mimics group; miR-221-5p, Over-expression of miRNA-221-5p group. ^**^p < 0.01 versus negative mimics group

**Fig. 5:**
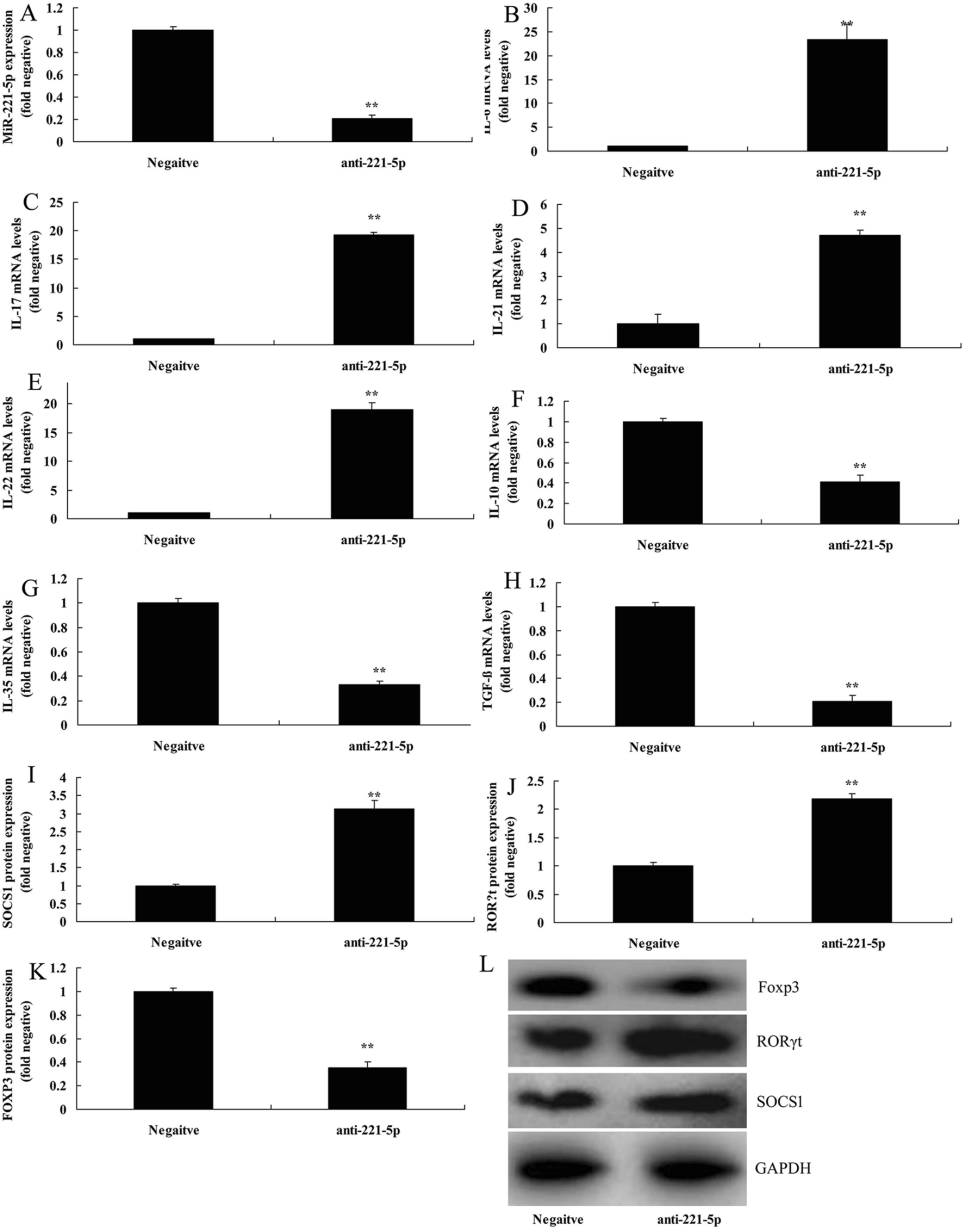
Down-regulation of miRNA-221-5p regulates imbalance of Th17 and Treg Cells in model asthma through RORγt/Foxp3 by Targeting SOCS1. The expression of miRNA-221-5p (**A**), IL-6 (**B**), IL-17 (**C**), IL-21 (**D**), IL-22 (**E**), IL-10 (**F**), IL-35 (**G**) and TGF-β (**H**) levels, SOCS1, RORγt and Foxp3 protein expression by statistical analysis (**I**–**K**) and western blotting analysis (**L**). Negative, negative mimics group; anti-221-5p, down-regulation of miRNA-221-5p group. ^**^p < 0.01 versus negative mimics group

In the present study, we investigated the effects of miRNA-221-5p on Treg and Th17 cells ratio in asthma. The expression of miRNA-221-5p was increased in model of asthma, compared with negative group (Fig. 2A) The *vitro* model of asthma treated with miRNA-221-5p mimic resulted in the reduction of IL-6, IL-17, IL-21 and IL-22 levels, and induction of IL-10, IL-35 and TGF-β levels. (Fig. 2B–H). Additionally, heat map and network signaling pathway showed that miRNA-221-5p regulated SOCS1, RORγt and Foxp3 signaling pathway (Fig. 3A–C). The 3’UTR of SOCS1 showed potential alignment with miRNA-221-5p sequence and luciferase reporter activity levels were enhanced following over-expression of miRNA-221-5p, in comparison with negative group (Fig. 3D–E). Over-expression of miRNA-221-5p suppressed the protein expression of SOCS1 and RORγt and induced that of Foxp3 in in vitro model of asthma, compared with negative group (Fig. 4A–D). Over-expression of miRNA-221-5p suppressed the protein expression of SOCS1 in in vitro model of asthma, in comparison with negative group (Fig. 4E). Then, miRNA-221-5p mimics was used to reduce miRNA-221-5p expression in model of asthma, compared with negative group (Fig. 5A). Down-regulation of miRNA-221-5p increased the levels of IL-6, IL-17, IL-21 and IL-22, and reduced those of IL-10, IL-35 and TGF-β in in vitro model of asthma, compared with negative group (Fig. 5B–H). Down-regulation of miRNA-221-5p induced the protein expression of SOCS1 and RORγt and suppressed that of Foxp3 in in vitro model of asthma, in comparison with negative group (Fig. 5I–L).

### The inhibition of SOCS1 attenuated the effects of anti-miRNA-221-5p on Th17/Treg ratio in asthma

**Fig. 6:**
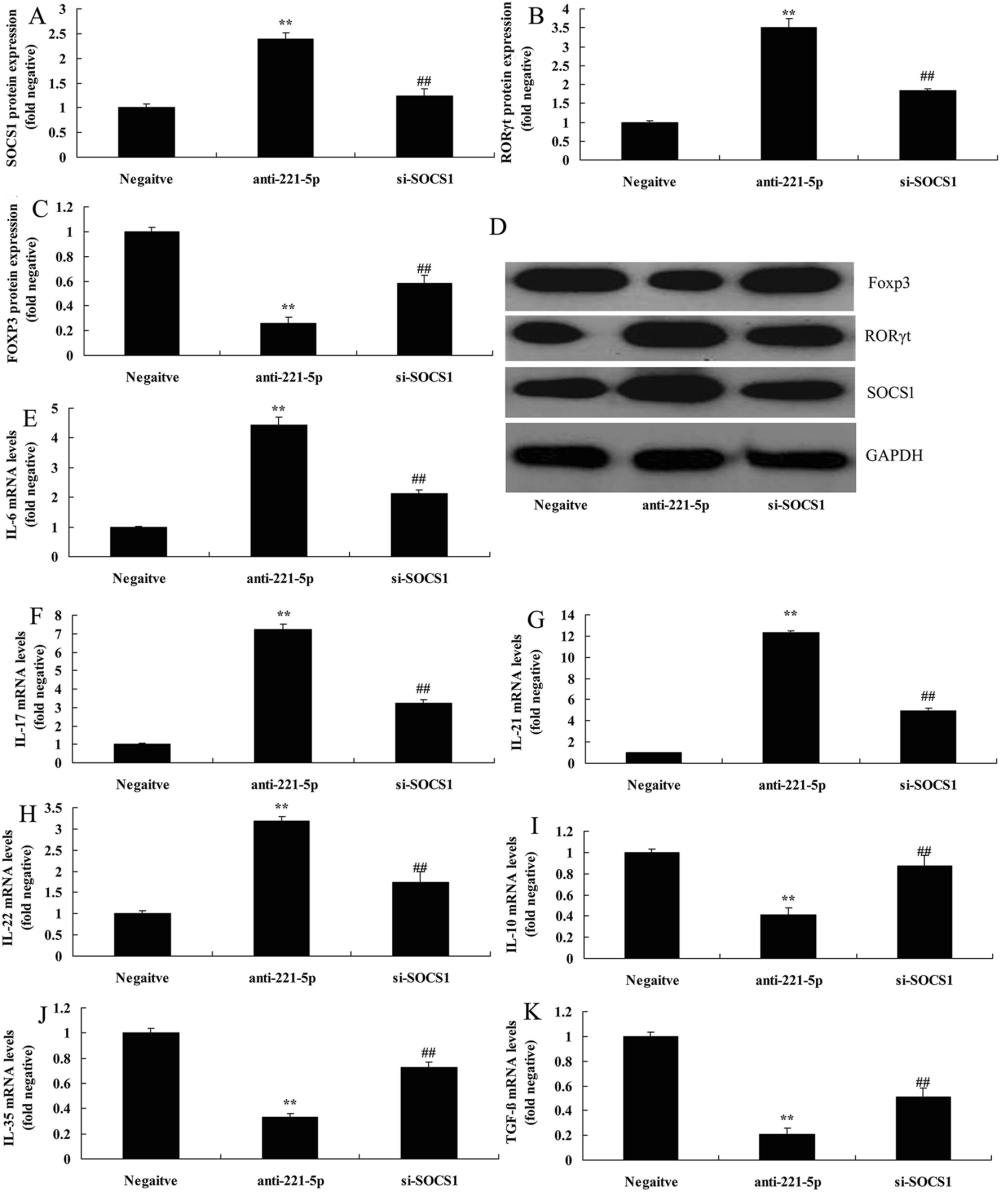
The inhibition of SOCS1 reduced the effects of anti-miRNA-221-5p on Th17/Treg Ratio in asthma. SOCS1, RORγt and Foxp3 protein expression by statistical analysis (**A**–**C**) and western blotting analysis (**D**), IL-6 (**E**), IL-17 (**F**), IL-21 (**G**), IL-22 (**H**), IL-10 (**I**), IL-35 (**J**) and TGF-β (**K**) levels. Negative, negative mimics group; anti-221-5p, down-regulation of miRNA-221-5p group; si-SOCS1, si-SOCS1 and down-regulation of miRNA-221-5p group. ^**^p < 0.01 versus negative mimics group, ^##^p < 0.01 versus down-regulation of miRNA-221-5p group

To examine the role of SOCS1 in the effects of anti-miRNA-221-5p on Th17/Treg ratio in asthma, si-SOCS1 was used to decrease the effects of anti-miRNA-221-5p on Th17/Treg ratio in asthma. The administration of si-SOCS1 suppressed the protein expression of SOCS1 and RORγt and induced that of FOXP3 in in vitro model of asthma following anti-miRNA-221-5p, in comparison with anti-miRNA-221-5p group (Fig. 6A–D). *The inhibition of SOCS1 attenuated the effects of anti-miRNA-221-5p on* the increased levels of IL-6, IL-17, IL-21 and IL-22, and suppressed levels of IL-10, IL-35 and TGF-β in in vitro model of asthma following anti-miRNA-221-5p, compared with anti-miRNA-221-5p group (Fig. 6E–K).

### The inhibition of RORγt attenuated the effects of anti-miRNA-221-5p on Th17/Treg ratio in asthma

**Fig. 7:**
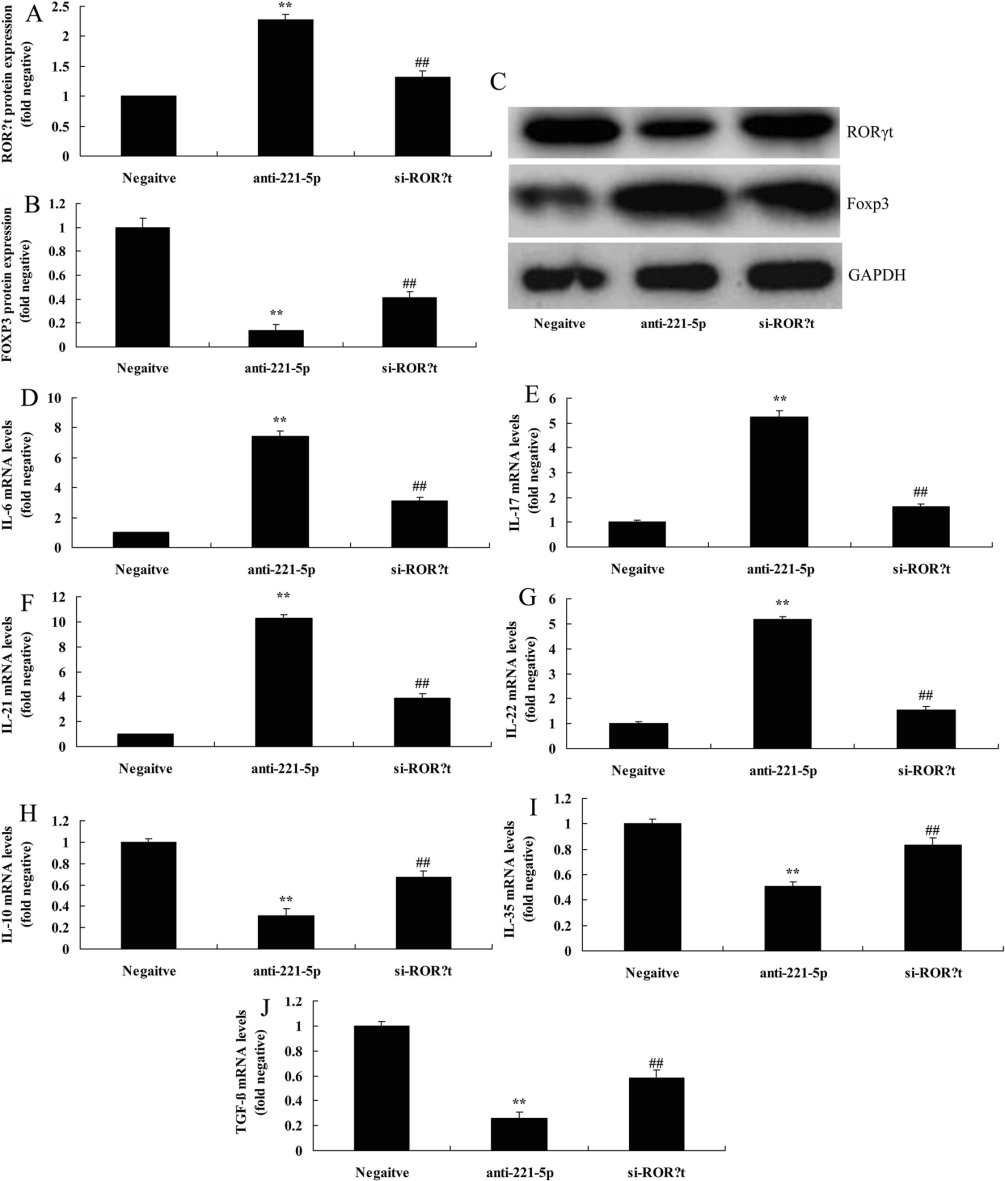
The inhibition of RORγt reduced the effects of anti-miRNA-221-5p on Th17/Treg Ratio in asthma. RORγt and Foxp3 protein expression by statistical analysis (**A** and **B**) and western blotting analysis (C), IL-6 (D), IL-17 (E), IL-21 (**F**), IL-22 (**G**), IL-10 (**H**), IL-35 (**I**) and TGF-β (**J**) levels. Negative, negative mimics group; anti-221-5p, down-regulation of miRNA-221-5p group; si-RORγt, si-RORγt and down-regulation of miRNA-221-5p group. ^**^p < 0.01 versus negative mimics group, ^##^p < 0.01 versus down-regulation of miRNA-221-5p group.

To explore the function of RORγt in the effects of anti-miRNA-221-5p on Th17/Treg Ratio in asthma, si-RORγt was used suppress the protein expression of RORγt and induced that of FOXP3 in in vitro model of asthma following anti-miRNA-221-5p, compared with anti-miRNA-221-5p group (Fig. 7A–C). The inhibition of RORγt also attenuated the effects of anti-miRNA-221-5p on the increased levels of IL-6, IL-17, IL-21 and IL-22 levels, and inhibited levels of IL-10, IL-35 and TGF-β in in vitro model of asthma following anti-miRNA-221-5p, in comparison with anti-miRNA-221-5p group (Fig. 7D–J).

### The inhibition of FOXP3 attenuated the effects of miRNA-221-5p on Th17/Treg ratio in asthma

**Fig. 8:**
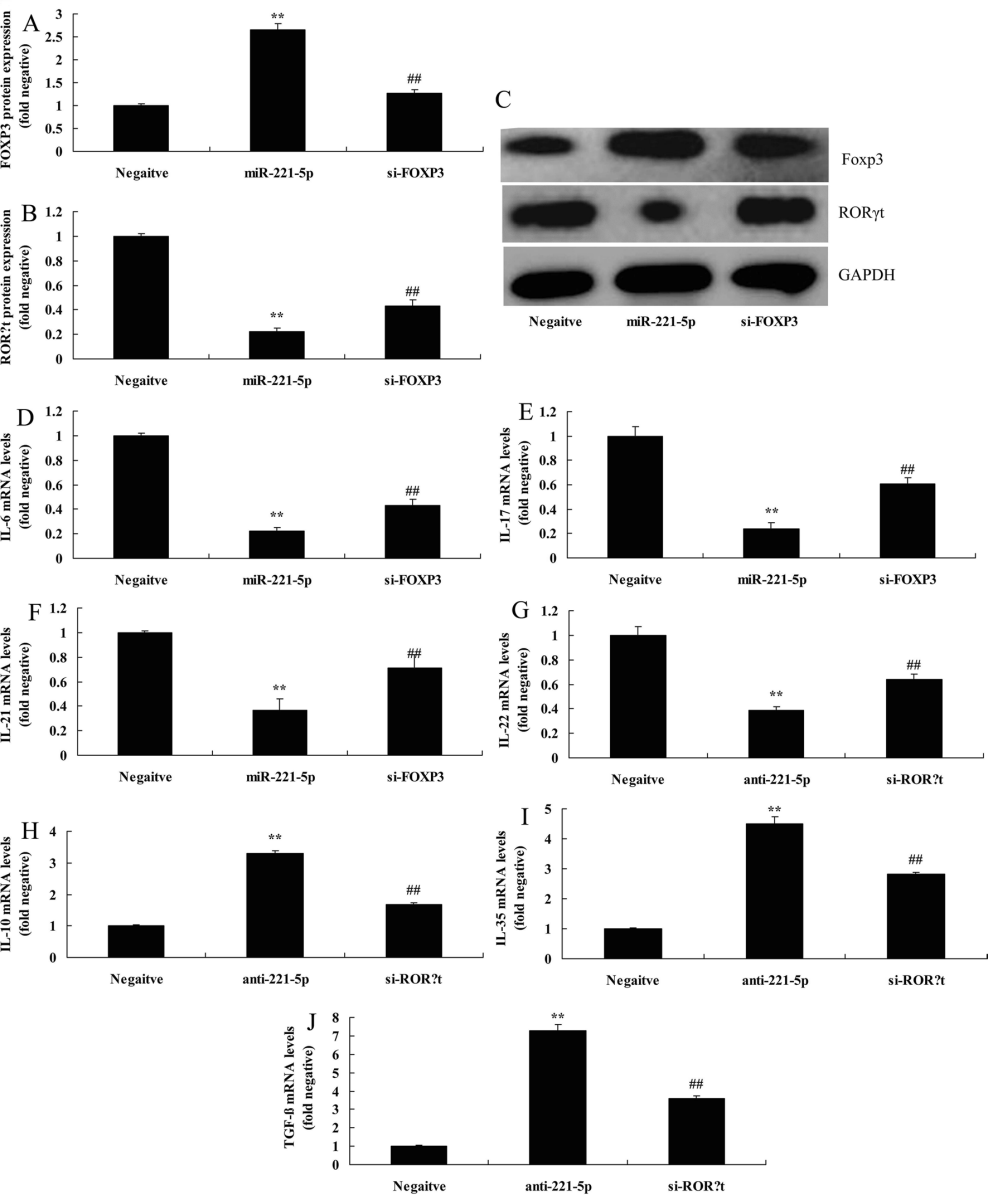
The inhibition of FOXP3 reduced the effects of miRNA-221-5p on Th17/Treg Ratio in asthma. RORγt and Foxp3 protein expression by statistical analysis (**A** and **B**) and western blotting analysis (**C**), IL-6 (**D**), IL-17 (**E**), IL-21 (**F**), IL-22 (**G**), IL-10 (**H**), IL-35 (**I**) and TGF-β (**J**) levels. Negative, negative mimics group; anti-221-5p, down-regulation of miRNA-221-5p group; si-FOXP3, si-FOXP3 and down-regulation of miRNA-221-5p group. ^**^p < 0.01 versus negative mimics group, ^##^p < 0.01 versus down-regulation of miRNA-221-5p group

To investigate the role of FOXP3 in the effects of miRNA-221-5p on Th17/Treg ratio in asthma, si-FOXP3 was employed to suppress the protein expression of FOXP3 and induced that of RORγt in in vitro model of asthma following miRNA-221-5p, compared with miRNA-221-5p group (Fig. 8A–C). The inhibition of FOXP3 attenuated the effects of miRNA-221-5p on the inhibition of IL-6, IL-17, IL-21 and IL-22 levels, and promotion of IL-10, IL-35 and TGF-β levels in in vitro model of asthma following anti-miRNA-221-5p, in comparison with anti-miRNA-221-5p group (Fig. 8D–J).

## Discussion

Bronchial asthma is a common chronic allergic disease of the airway in the respiratory system, which is a serious threat to human health characterized by its diffuse, obstructive, paroxysmal, and reversible properties [1]. The prevalence of bronchial asthma has increased year by year due to the influence of genetics, urbanization, population intensiveness, air pollution caused by industrialization [10]. In this study, we identified that miRNA-221-5p expression was reduced in vivo model of asthma. Zhang et al. showed that miR-221-3p expression was significantly decreased in subjects with asthma [9]. This study mainly used PBMCs for vitro model of asthma, and this is an insufficient of this study. We should more model or morel CD cell or immune cell to verify our conclusion in further research.

miRNAs are endogenous non-coding single-stranded small-molecule RNAs that regulate the normal growth and development of the body as well as the occurrence of diseases by suppressing or degrading target genes. miRNAs have been validated to play key roles in the pathogenesis of asthma, either inhibiting or enhancing its development, which may serve as therapeutic targets for asthma in the future [11]. These results of these results were expected that over-expression of miRNA-221-5p reduced IL-6, IL-17, IL-21 and IL-22 levels, and increased IL-10, IL-35 and TGF-β levels in vitro model of asthma. Zhao et al. reported that downregulation of miR-221 occurs in spinal cord I/R injury and regulates the inflammatory response and apoptosis of neuronal cells through its impact on TNFAIP2 [12]. This study only analyzed the effects of miRNA-221-5p regulates IL-6, IL-17, IL-21, IL-22, IL-10, IL-35 and TGF-β levels, and which was a sufficient for present research. We will analyzed the effects of miRNA-221-5p regulates other cytokines, such as IL-1β, TNF-α, IL-12 and so on.

Asthma is a chronic inflammatory response involving interactions of multiple inflammatory cells, participation of many mediators and cytokines. The pathogenesis of asthma is associated with immune responses [13]. Immunological studies suggest that helper T cells, such as Th1, Th2, and Th17 play important roles in the occurrence and progression of asthma [13]. Among them, the imbalance of Th1/Th2 and Th17/Treg is closely associated with the pathogenesis of asthma [14, 15]. Our findings provide strong evidence that the down-regulation of miRNA-221-5p increased IL-6, IL-17, IL-21 and IL-22 levels, and reduced IL-10, IL-35 and TGF-β levels in vitro model of asthma. Asquith et al. determined that miR-221 regulates Th1/Th17 cytokine production in nonhuman primates [16].

Th17 is a novel IL-23-dependent CD4+ T cell, which was first found in the animal models concerning autoimmune encephalomyelitis (EAE) and collagen-induced arthritis [4]. The main function of Th17 cells is to produce various cytokines, such as IL-17, IL-21 and IL-22, which stimulate epithelial cells, fibroblasts and smooth muscle cells of the airway to secrete CXCL1 and CXCL8, thereby participating in autoimmune diseases [17]. Therefore, Th17 and its secreted inflammatory factors are involved in the immune regulatory process. Therefore, Th17 and its secreted inflammatory factors are involved in the immune regulatory process. We found that the inhibition of RORγt reduced the effects of anti-miRNA-221-5p on Th17/Treg Ratio in asthma.

The pathological basis of bronchiolitis is that RSV stimulates airway epithelial cells, causing a series of inflammatory reactions, including bronchiole obstruction, airway hyper-responsiveness and airway remodeling. A large number of studies have confirmed that Th17/Treg imbalance plays an important role in the immune mechanism of bronchiolitis [18]. At present, Th17 has confirmed to be differentiated from the natural T cell precursor Th0. Under the stimulation of specific antigen and multiple signals, Th0 can be differentiated into different T helper cell subpopulations. Under the combined action of transforming growth factor β (TGF-β) and IL-6, naive T cells could be induced to differentiate into Th17 cells, to subsequently secrete IL-17, and to express ROR-γt, while ROR-γt is a key transcription factor of Th17 cell differentiation [19, 20]. This study showed that the inhibition of FOXP3 reduced the effects of miRNA-221-5p on Th17/Treg Ratio in asthma. Khalifa et al. reported that miR-222 regulates FoxP3 polymorphism rs2232365A/G in rheumatoid arthritis [21].

SOCS3 can bind to specific cytokine receptors and inhibit the JAK/STAT signaling pathway, while RSV virus suppresses the interferon-mediated antiviral response by affecting the protein expression of SOCS1/3, both of which are also considered to be important factors in the anti-RSV infection and acutely exacerbated asthma. Therefore, the effects of RORγt and SOCS1/3 acutely exacerbated RSV infection of asthma Our findings confirmed that Over-expression of miRNA-221-5p suppressed SOCS1 and RORγt protein expression and induced Foxp3 protein expression in vitro model of asthma. The inhibition of SOCS1 reduced the effects of anti-miRNA-221-5p on Th17/Treg Ratio in asthma. Liu et al. showed that lncRNA GAS5 reverses metastasis by targeting miR-221/SOCS3 in pancreatic cancer [22]. Meanwhile, the effect of miRNA-221-5p on Th17/Treg Ratio in asthma, whether miRNA-221-5p regulates CD4 T cell in cancer model, and we will analyze the anti-cancer or pro-cancer effects of miRNA-221-5p on tumour immune in further research.

## Conclusion

**Fig. 9:**
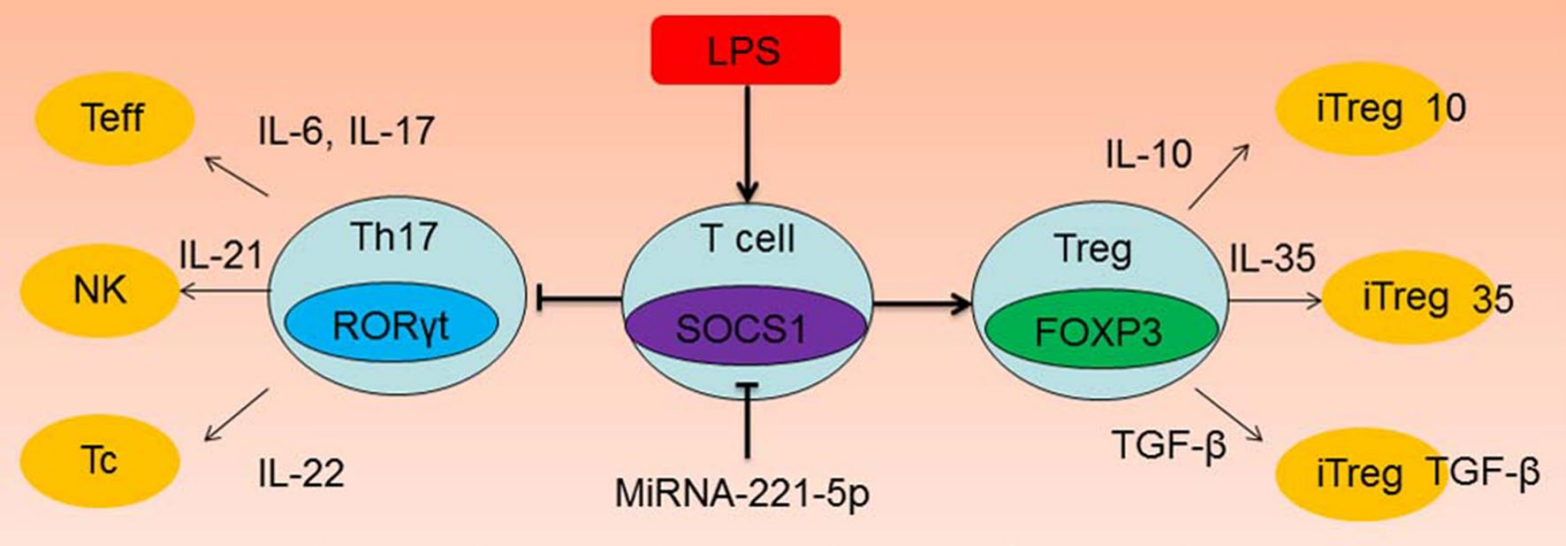
MiRNA- Regulates the Th17/Treg Ratio in asthma via RORγt/Foxp3 by Targeting SOCS1

In conclusion, miRNA-221-5p may play critical roles in driving the differentiation of Th17/Treg ratio via RORγt/Foxp3 by Targeting SOCS1, reduced the function of Th17 cells by directly inhibiting RORγt/SOCS1 and promoted the function of Treg cells via Foxp3/ SOCS1 in asthma (Fig. 9). These results, speculations and the results of future studies may serve to implicate miRNA-221-5p/Th17/Treg ratio as a possible new therapeutic target for asthma.

## Data Availability

The datasets used or analyzed during the current study are available from the corresponding author on reasonable request.

## Abbreviations

SOCS: Cytokine signaling
OD: Optical density
H&E: Hematoxylin and eosin
cDNA: Complementary Deoxyribose Nucleic Acid
PBMCs: Peripheral blood mononuclear cells
RPMI: Roswell Park Memorial Institute
LPS: Lipopolysaccharides
UTR: Untranslated region
SPSS: Statistical Product and Service Solutions
ANOVA: One-way analysis of variance
EAE: Encephalomyelitis
